# Pollen-Food Allergy Syndrome: A not so Rare Disease in Childhood

**DOI:** 10.3390/medicina55100641

**Published:** 2019-09-26

**Authors:** Carla Mastrorilli, Fabio Cardinale, Arianna Giannetti, Carlo Caffarelli

**Affiliations:** 1 Pediatric and Emergency Operative Unit, Policlinic Consortium University Hospital-Pediatric Hospital “Giovanni XXIII”, 70126 Bari, Italy; carla.mastrorilli@icloud.com (C.M.); fabiocardinale@libero.it (F.C.); 2 Pediatric Clinic, University Children’s Hospital, Medicine and Surgery Department, University of Parma, 43100 Parma, Italy; 3 Pediatric Unit, Department of Medical and Surgical Sciences, University of Bologna, 40138 Bologna, Italy; arianna.giannetti@libero.it

**Keywords:** children, component resolved diagnostics, food allergy, oral allergy syndrome, pollen, pollen-food allergy syndrome, seasonal allergic rhinitis

## Abstract

Seasonal allergic rhinoconjunctivitis (SAR) affects millions of people worldwide, particularly in childhood and adolescence. Pollen food allergy syndrome (PFAS) is a common adverse reaction occurring few minutes after the consumption of vegetable foods in patients with pollen-induced SAR. PFAS has rarely been investigated in the pediatric population, as it has been mainly examined as an adult disease. Recent studies suggested that PFAS might be more frequent in childhood than previously recognized. The present review aims to give an overview of the epidemiology, pathophysiology, diagnosis, management and prognosis of PFAS in children with SAR-induced by pollens.

## 1. Introduction

Pollen-food allergy syndrome (PFAS) is a common immunoglobulin E (IgE)-mediated allergic disease caused by a cross-reaction among pollens and vegetable foods [1]. The pathogenesis of PFAS is linked to a respiratory allergy to pollens and subsequent cross-reactivity between pollens and homologous epitopes contained in plant-derived food proteins (class 2 food allergy) [2]. PFAS is clinically characterized most frequently by isolated oral and pharyngeal symptoms at immediate onset following food intake, which is called oral allergy syndrome (OAS) [3]. OAS was firstly described in 1942 among patients affected by pollinosis and related to labile allergens in fresh vegetables. Nowadays, the term PFAS has replaced the traditional OAS, that may be unclear [4]. In fact, although rarely several studies described systemic symptoms, such as urticaria, nausea, vomiting and anaphylaxis among patients with OAS [5,6]. Therefore, OAS may represent the clinical expression not only of sensitization to cross-reacting food allergens but also of primary sensitization to genuine food allergens. OAS could be triggered by any food source or any food allergen [7]. Furthermore, it can be the first symptom or a concomitant manifestation of a systemic allergic reaction (class 1 food allergy) [8]. This review focuses on characteristics of PFAS in children. The literature search was undertaken in April 2018 and it was limited to papers published in the last 10 years in English language. No type of study was preferred. Earlier articles were selected by references of selected articles or they were known by their authors. We also assessed studies in adults that were helpful for the purpose of this review.

## 2. Epidemiology

The prevalence of food allergies has increased in the recent past, and the diagnostic criteria have been well recognized [9]. PFAS is considered the most frequent food allergy in adults and the typical comorbidity of pollinosis [10]. However, PFAS is observed also in children who suffer from pollen-induced seasonal allergic rhinoconjunctivitis (SAR) [11], starting at preschool age, with a prevalence steadily growing with age [12] (Table 1). PFAS can occur at any time of year [9]. It is unclear whether incidence and severity of symptoms increase during pollen allergy seasons [2,13]. A real prevalence of PFAS has not been yet defined because of the controversial definition of PFAS and OAS, underreporting mild symptoms and patient food avoidance [14]. Furthermore, the lack of studies performing oral challenges with triggering foods in children cannot permit to acquire clinical information about the frequency and severity of OAS. In the first studies about OAS [15], 45% of 80 highly atopic patients reported oral symptoms after food intake. Along this line, in Korea, the prevalence of OAS in children with pollen allergy was 42.7% [11] and in Swedish children was 25%, with 31% of symptoms among patients allergic to birch (vs 5% among those sensitized to other pollens) [15]. On the contrary, in Mexico, the prevalence of OAS among children with pollen allergy was around 9.6–12.2% depending on the type of pollen [16]. Regarding PFAS, Dreborg and Eriksson showed in large adult and pediatric studies that over 70% of patients with birch allergy had symptoms of PFAS and 20% of those allergic to grass and mugwort [17,18]. A survey of North American allergists reported a prevalence of only 5% in children with pollinosis [19], as a consequence of physician underdiagnosis and patient underreporting. In Italy, the incidence was 29% in children with seasonal allergic rhinoconjunctivitis (SAR) [20]. Croatian children with SAR had PFAS in 29.7% of cases [21]. Recent pediatric studies from single clinics in UK and Australia showed a prevalence of 48% and 12.9%, respectively [13,22]. Overall, the frequency of PFAS in childhood is higher than previously recognized, and similar to that in adults [23]. The prevalence of PFAS is influenced by local diet, and regional prevalence of atopic diseases [22,24]. Risk factors for PFAS in children are female gender [12,13], older age [12,13], parents affected by atopic diseases [11], overweight [12], and environmental tobacco smoke exposure [12]. Regional distribution of pollen taxa [20] may also play a role. For instance, in Mexican children PFAS is associated with sensitivity with pollens from the *Quercus* species [16].

## 3. Pathogenesis

**Figure 1:**
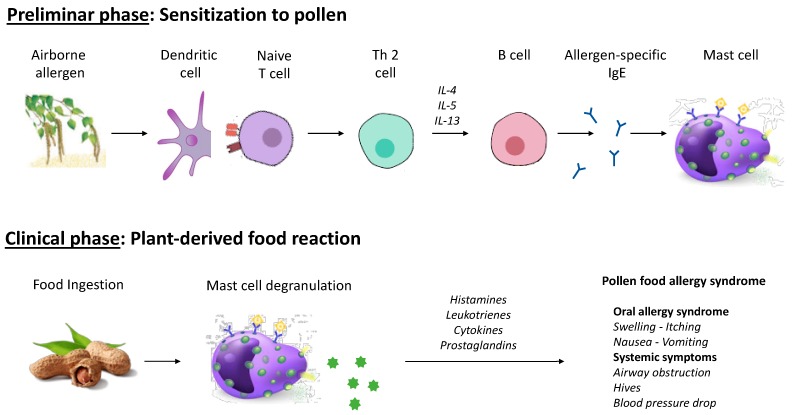
Time course, pathogenesis, and manifestations of pollen-food allergy syndrome. (**A**) **Preliminar phase:** Sensitization to pollen. Airborne allergen contact via the respiratory tract activates, after processing by antigen presenting cells (APC), such as dendritic cell, naïve T helper cells with the antigen-specific receptors. In allergic sensitization the differentiation leans towards the TH2 phenotype, with the release of interleukins 4, 5, and 13. Eosinophils are recruited and activated by IL-5. The production of antigen-specific IgEs through B cells (primary sensitization) are stimulated by IL-4 and IL-13. (**B**) **Clinical phase:** Plant-derived food reactions. Ingestion of cross-reacting molecules induces the secondary immune response via T-cell and IgE memory. The degranulation of mast cells leads to the release of granules of preformed inflammatory mediators (e.g., histamine) and the de novo synthesis and/or release of inflammatory mediators (e.g., Leukotrienes), proteases (e.g., Tryptase), cytokines (e.g., IL-4) and chemotactic molecules. Allergic symptoms occur shortly after ingestion in the locations of the contact to the allergen (e.g., mouth, throat, intestine) or in the organs (systemic symptoms). IgE, immunoglobulin E; IL, interleukin.

PFAS occurs in patients previously sensitized to pollens. The contact via the respiratory tract to pollen allergens leads to the allergen sensitization process and the development of allergic rhinitis. Then, in a second step upon exposure to raw plant-derived foods that contain cross-antigens to the inhaled allergens, IgE antibody to pollen cross-react to plant-derived protein (Figure 1) [25]. If two or more allergenic proteins resemble showing the same or similar epitopes, IgE antibodies may cross-react binding to the other allergen, also without prior contact and sensitization [26]. Over 70% identity in primary sequence of pollen and food proteins is generally required for cross-reactivity [27]. Clinical cross-reactions to foods occurs only in a subset of children sensitized to pollens; some patients react to one specific food, although others to several foods [27]. Generally, the plant-derived proteins are sensitive to heat exposure, gastric acid and digestive enzymes, and induce mild manifestations at the oral mucosa (OAS), as they lose their allergenic properties in the digestive tract. The same foods (e.g., apple sauce) are typically tolerated after cooking. These food allergens (called class 2 food allergens) differ from class 1 food allergens that are able to sensitize through the gastrointestinal tract or the skin [28,29] and elicit allergic reactions. Factors that determine the occurrence of clinical hypersensitivity reactions in sensitized children are complex and linked to the immune system of the host [30] and the main characteristics of the allergen (e.g., lability after digestion and heat).

## 4. Triggering Foods

The number of foods triggering PFAS is continuously growing (Table 2). The regional dissimilarities concerning which foods are responsible for PFAS mainly depend on prevalence of grasses and weeds in different areas of the world, as well as the foods consumed [22]. In Northern European countries, many patients with birch tree pollen sensitization develop PFAS that is induced by plant food, such as tree nuts and fruits, particularly those of the *Rosaceae* family [24]. Conversely, in Southern European countries, PFAS is mainly represented in patients polysensitized to various pollens [31,32]. Fruits such as apples, peaches, cherries, and vegetables such as celery, carrots, and tomatoes, are some of the foods more frequently associated with PFAS. Walnuts and hazelnuts have also been implicated. Moreover, soybean or soymilk-induced OAS and anaphylaxis have been often reported. In Japan, sensitization to Japanese cedar is the main trigger of airborne allergy, and it’s associated with PFAS due to fresh tomato in some cases [33]. Also, apple allergy among alder pollen sensitized patients are reported [34]. In Australian children, watermelon is the most common triggering food among children with grass pollen and birch allergy [22]. Pineapple (*Ananas comosus*) has been shown the main food eliciting PFAS in Mexican children sensitized most frequently to oak and ash tree [16]. Kiwifruit, followed by peach, is the fruit most frequently involved in OAS among Italian children with SAR-induced mainly by grass pollen, olive tree and plantain [12]. 

## 5. Clinical Features

Symptoms of PFAS are generally restricted to the oropharyngeal mucosa and occur immediately, or in 5–10 min after consuming fresh fruits, vegetables, nuts, legumes and seeds. Oropharyngeal symptoms comprise labial and oropharyngeal pruritus, paresthesia, angioedema of the oral mucosa, lips, tongue, palate and pharynx, itchy ears, mucosal red patches and blisters and sensation of laryngeal tightness that may cause hoarseness (OAS). Symptoms typically last for a few minutes to half an hour. Systemic reactions have been reported in 2–10% of cases. They include nausea, abdominal discomfort, diarrhea, rhinitis, difficulty in breathing, skin rash, urticaria-angioedema or hypotension, and anaphylaxis in 1–2% [38,39]. Now, no diagnostic method is able to distinguish children who develop systemic reactions. So, it is important to underline that OAS can be the first symptom of a more severe reaction [16,40]. Some patients refer to tolerate some varieties of the food. This may be the result of different protein content.

Many pan-allergens linked to OAS, such as PR-10–like and profilin, are labile to heat and digestion. Therefore, patients frequently tolerate cooked fruits or vegetables which provoke OAS [41,42]. Different features are shown for a few foods, most notably tree nuts and peanuts, due to protein’s stability during cooking [42]. In case of systemic reactions, it is suggested a strict avoidance of the offending foods in all formulas.

## 6. Diagnostic Evaluation

Clinical history can identify PFAS with high sensitivity and specificity and represents the main diagnostic item [24,43]. Oral food challenge (OFC) is the definitive mean for ascertaining food allergy. Nevertheless, OFC in PAFS is hampered by different allergen levels in cultivars. Double-blind placebo-controlled food challenge that represents the gold standard for detecting food allergy, is difficult to perform since the fresh material is labile, it must be masked and should not be quickly swallowed to permit the development of the reaction. So, this difficulty could be solved with the procedure of recombinant food allergens that are standardized [25]. The decision of performing OFC should consider that negative and positive predictive values of clinical history of OAS was 100% and 92% in comparison with OFC [44]. Furthermore, one should consider the degree of symptoms, nutritional requirements, food habits and preferences. Therefore, a diagnosis based on history and skin prick tests (SPT) or serum IgE to food and inhalant allergens, is widely supported in specific clinical situations. Many Authors think that a convincing clinical history of respiratory symptoms linked to pollen exposure and immediate symptoms following cross-reacting food ingestion and positive IgE tests to the relevant allergens are enough to guarantee the diagnosis of OAS. OFC is mandatory when history is unclear and systemic reactions occurred [1]. Patients with positive history and negative IgE tests should also undergo OFC with fresh food.

### Skin Prick Tests

The identification of aeroallergen sensitization and cross-reactive patterns can be assessed by SPTs. Besides, the absence of pollen sensitization can indicate a different etiology. Regarding vegetables and fruits, plant-derived proteins are often labile and denaturized during extraction for commercial preparation. Then, the use of natural extracts for in vitro or in vivo diagnostic assays are frequently disappointing, since they assessed only sensitization to stable allergens. It has been extensively documented that sensitivity and specificity of SPTs with fresh fruit using the prick-by-prick technique are higher than those of SPTs using commercial extract. This technique consists of pricking firstly the fresh foods with lancet and then to the skin, and it is contemplated as the standard for identifying vegetable foods sensitization [45,46]. It is possible to use freezing fruits up to 2 years, since that does not alter the antigenic characteristics [47]. So, an appropriate and more suitable substitute to fresh fruit for SPT can be represented by frozen fruits. IgE tests to food or inhalant allergens instead of SPT can be necessary in children with dermographism, extensive skin disease, interfering drug use. However, false positive results may result from cross-reactivity with pollen allergens or false negative ones for a loss of allergenicity during extract preparation. Patch tests have no diagnostic value [48].

## 7. Molecular Assay

Component-resolved diagnostics (CRD) represents a reliable instrument in the diagnosis of PFAS, as it offers the opportunity to establish and compare individual sensitization profiles based on the cross-reactive proteins. However, only few allergens belonging to class 1 and class 2 have been cloned, sequenced, and expressed as recombinant proteins. The principal pan-allergens comprise three protein families: profilins, pathogenesis-related protein type 10 (PR-10), and non-specific lipid transfer proteins (LTP, PR-14). PR-10s are the main cause of PFAS displaying with oral symptoms in Northern and Central Europe, where pollen allergy is predominantly related to birch and alder pollens and symptoms are mostly triggered by PR-10-containing *Rosaceae* (e.g., apple, peach). So, in Northern countries PFAS represents a distinct disease with a well-known pathogenesis. On the contrary, the molecules involved in PFAS are various among Mediterranean countries, characterized by several pollens distributed during the year [8]. Moreover, patients with SAR are frequently pollen-polysensitized and the etiologic diagnosis could be complex, making it hard to perform a classification of PFAS in Southern Europe.

## 8. Pathogenesis Related Protein Type 10

The family of PR-10-like proteins, comprising the primary sensitizer Bet v 1 from silver birch pollen, have a shared tertiary structure with seven antiparallel beta sheets, a long c-terminal and alpha helix and two short alpha helices [49,50,51]. The sequences of the proteins are highly similar and Bet v 1 shares a molecular homology with pollens of several plants (e.g., hazel, hornbeam, and hop-hornbeam) [49], in fruits, nuts and seeds. Fruits associated with PFAS are in the *Rosaceae* fruits (e.g., apple (Mal d 1), cherry (Pru av 1), apricot (Pru ar 1), pear (Pyr c 1)), the *Apiaceae* vegetables (e.g., carrots (Dau c 1), celery (Api g 1)), and hazelnut (Cor a 1)) [50]. Since PR-10 (or Bet v 1 homologous) proteins are denatured and destroyed after digestion and heating processes, symptoms are restricted to oral-pharyngeal area not progressing to systemic [51]. Numerous Northern European studies revealed birch pollen as a main trigger of OAS with the offending food represented primarily by apple [52,53]. Oak and ash tree (*Quercus sp.* and *Fraxinus sp*.), but not birch nor olive tree, were the pollen linked with the PFAS in Mexico [16]. A particular case of Bet v 1 homologous is represented by soybean Gly m 4, that is reported to induce systemic symptoms and is considered as a marker of severe soybean allergy [33].

## 9. Profilins

Profilins are small (12–15 kDa) proteins, that share more than 75% of sequences with each other [54]. They are ubiquitously spread in eukaryotic cells and can, therefore, be found in pollens, plant-derived foods and in latex. Profilins are involved in many essential cellular processes, including cell motility, membrane organization and signaling pathways. Since they present a homologous structure, profilin-specific IgE might cross-react with homologues from almost all plant source. Profilin is considered a minor allergen, since allergic individuals can be sensitized in 5 to 40% of cases, it is quite frequent (>15%) since preschool (<6 years) age and its rate increases with age and disease duration [55]. Furthermore, IgE sensitization to profilin represents a risk factor in case of multiple pollen and food allergens allergic reactions [55]. Many pollens may be the primary sensitizer in patients sensitized to profilin, such as grass, birch, ragweed, mugwort, and olive [56]. However, its clinical relevance as respiratory allergen remains still debated [57]. Since profilins are labile to gastric enzymes and heat denaturation, profilin-sensitized patients after ingesting of raw foods usually develop OAS reactions. In clinical settings, allergy to melon, watermelon, citrus fruits, tomato, and banana have been proposed as a marker of profilin in adults [58]. In childhood, allergic cross-reactions mediated by profilins involve *Cucurbitaceae* (melon, watermelon, and cucumber), tomato, peach, banana, and kiwifruit [12]. In addition, cross-reactions mediated by profilins have been defined with exotic fruit, such as lychee and pineapple.

## 10. Lipid Transfer Proteins

Non-specific lipid transfer proteins (LTPs), named for their capability to bind and transfer lipid molecules across membranes in vitro, such as cutin and suberin, comprise a family of 7 kDa (LTP 2 subfamily) or 9 kDa (LTP 1 subfamily) proteins. According to their role, LTPs are placed in the peel of fruits more than in the pulp. LTPs belong to the type 14 of pathogenesis-related (PR) proteins, and act in plant protection with antifungal and antibacterial activities, triggered by environmental stress, pathogen infection, and antibiotic stimuli [59]. Allergenic LTPs have been recognized in tree and weed pollens, in vegetable food allergen sources, and in latex. Nevertheless, the LTP hypersensitivity may be expressed clinically in a variable way, with many patients that tolerate foods being strongly sensitized to it, others that react only if associated with co-factors, including exercise, nonsteroidal anti-inflammatory drugs, or alcoholic beverages, and subjects experiencing severe allergic reactions despite low specific IgE levels. The principal sensitizer in most patients allergic to LTPs is represented by the peach, since it typically produces symptoms as a first food, and IgE levels to Pru p 3 are generally higher than other LTPs [60]. However, the precise way of sensitization to LTPs is yet unknown, since the pollen of *Artemisia* and *Platanus* has been considered a marker or an inducer [61].

People allergic to LTP, co-sensitized to profilin and/or PR-10 showed a lower frequency of severe food adverse reaction [62] in an Italian study including 148 peach allergic adults. The profilin sensitization seems to have “protective” effects in reducing the manifestation of severe allergic reaction to food not only in adults sensitized to LTP, but also in those sensitized to seed storage proteins [63]. It must be underlined that these study populations comprised patients affected by both class 1 and class 2 food allergy. Considering pollen sensitization, it has been observed that patients with mild OAS reacted with greater frequency to pollens, both in terms of symptoms and specific IgE levels, in comparison with patients with severe OAS. This means that sensitization to LTP in the absence of pollinosis is a stronger sensitizer, although not linked to severe food allergic symptoms in patient with pollinosis. It may be dependent on a primary sensitization to a pollen not yet discovered. However, among children with pellitory allergy, one-quarter of Pru p 3-sensitized children reported anaphylaxis after ingesting LTP-containing foods, about half reported systemic allergic symptoms or oral allergy syndrome [64]. Further studies are needed to better explain the role of LTP as panallergen in childhood.

## 11. Endotypes of Pollen Food Allergy Syndrome

A molecular classification of PFAS has been determined among 1271 Italian children (aged 4–18 years) with SAR, establishing five distinct endotypes of PFAS [12], marked by peculiar profiles of IgE sensitization to panallergens. Endotypes of PFAS were identified with unsupervised cluster analysis and linked to three panallergen IgE sensitization, showing peculiar characteristics: “profilin PFAS endotypes” (sensitization to profilin), with symptoms elicited by *Cucurbitaceae*; “LTP PFAS endotype” (sensitization to LTP), living in Southern Italy, symptoms triggered by hazelnut and peanut; “PR-10 PFAS endotype” (sensitization to PR-10), with symptoms elicited by *Rosaceae*; “multi-panallergen PFAS endotype” (sensitization to ≥2 panallergens), recurrent allergic comorbidities and numerous culprit foods; “no-panallergen PFAS endotype” (no sensitization to any panallergen analyzed), mild disease and symptoms triggered by kiwifruit. These disease endotypes represent different and peculiar sensitization patterns, such as biomarkers of the main characteristics of PFAS, with the aim of the development of a tailored diagnostic and therapeutic approach in the clinical practice. However, it must be underlined about one-fourth of patients did not show a positive IgE response to any of the three panallergens. Therefore, larger studies are needed to analyze the biological mechanisms of IgE-mediated sensitization patterns and allergic multimorbidities.

## 12. Treatment Options

The treatment of PFAS is in the avoidance of raw triggering food. However, the management of PFAS is a matter of debate. A survey of allergists on OAS established that in 53% of cases complete avoidance of the triggering foods was recommended, 9% did not encourage food restrictions, and 38% proposed individual patient advices [19]. Since thermal processing of panallergens generally causes loss of their IgE binding ability, patients with PFAS usually tolerate cooked forms of the vegetable foods to which they are allergic, except for nuts. However, cooked food allergens keep their capacity to stimulate specific T cells [42]. Patients with atopic dermatitis and PFAS who eat cooked vegetable foods might have an exacerbation of their eczema because of activation of Bet v 1–specific T cells that migrate to the skin and stimulate effector responses [15].

About 4% of clinicians suggested avoiding potentially cross-reactive foods belonging to the same family [19,65]. It would be advisable to assess (by OFC) tolerance to other fruits of the same family, unless the patient is eating them without symptoms or IgE test results are negative.

OAS symptoms are unimportant and rapidly disappear. They generally do not need to be cured. In some cases, antihistamine anti-H1 can be administered. Severe allergic reactions with dyspnea or cardiovascular involvement should be treated with epinephrine. In selected cases, patients should carry an epinephrine autoinjector for reactions due to inadvertent ingestion of the culprite food.

There is no clear evidence that allergen immunotherapy (AIT) to foods may be helpful in OAS [1,66]. It has been described that regular ingestion of increasing amounts of fresh pollen-related food may induce clinical tolerance in patients with PFAS [67]. However, these results were not correlated with immunologic changes in food-tolerant subjects. AIT with recombinant Mal d 1 allergen in pollen-related apple allergic patients has been found to be safe and effective, inducing immune responses characteristic of peripheral tolerance [68]. Regarding AIT for systemic reactions to fruits and vegetables, only AIT to peanut has been shown to be effective. However, it greatly increases the rate of allergic and anaphylactic reactions in comparison with avoidance or placebo [67]. This limits its use in clinical practice. The efficacy of AIT to pollens on improving mucosal reactions to cross-reacting foods is unclear [1]. However, sublingual AIT to pollen allergens has been investigated with positive results in terms of T-cell tolerance, immune deviation, and regulatory T cells, as well as allergen-specific IgG4 [69,70,71]. Larger prospective controlled studies are warranted to clarify the issue.

## 13. Conclusions

PFAS represents a comorbidity of SAR induced by pollens, manifesting as adverse reaction to the ingestion of pollen-related foods. Its prevalence in childhood is higher than previously reported. Few studies investigated the main characteristics of PFAS in children. Although clinical history alone may ascertain precisely PFAS, OFC is considered the gold standard diagnostic tool in children with suspected food allergy. Furthermore, CRD is able in most of cases to understand the pathogenesis of the disease and determine individual endotypes based on the cross-reactive allergens. The therapeutic options of PFAS are still under discussion. Further studies are needed to analyze PFAS in childhood with the purpose of a tailored diagnostic and therapeutic approach of allergic multimorbidities.

## Figures and Tables

**Table 1:** Prevalence and main characteristics of pollen-food allergy syndrome #.

First Author, Year	Country	Prevalence, % ^§^	Patients, n	Age, Years	Sensitizing Pollens °	Triggering Foods *
Ma, 2003 [19]	USA	5	^^^	0–18	birch, ragweed	apple, banana, carrot
Westman, 2012 [15]	Sweden	25	2024	4–8	birch, grass, mugwort	−
Brown, 2014 [22]	Australia	14.7	163	4–17	grass, birch	watermelon, kiwi, banana
Ludman, 2015 [13]	UK	48	54	0–15	grass	hazelnut, apple, strawberry
Dondi, 2015 [20]	Italy	23.9	1360	4–18	grass, olive tree, plantain	kiwi, peach, hazelnut
Ivković-Jureković, 2015 [21]	Croatia	26.7	120	3.4–18.6	birch, grass, ragweed	apple, peach, carrot
Bedolla-Barajas, 2016 [16]	Mexico	8.9	267	6–14	oak, ash tree, Prosopis	pineapple, peach, avocado
Kim, 2018 [11]	Korea	42.7	300	0–18	birch, oak, mugwort	peach, apple, kiwi

# Clinical diagnosis from reported symptoms of oral allergy syndrome, ^§^ Study populations affected by hay fever, ° Most frequently involved sensitizing pollens * Most frequently involved triggering foods, ^^^ Data from a survey of allergists.

**Table 2:** Types of pollens involved in the pollen food allergy syndrome and the most frequent triggering foods [35,36,37].

Pollens	Triggering Foods		
	Fruits	Vegetables-Spices	Nuts-Seeds-Legumes
**Alder**	apple, cherry, peach, pear	parsley, celery	almond, hazelnut
**Birch**	kiwi, apple, pear, plum, apricot, cherry, tomato	celery, carrot, fennel, potato, green pepper, cumin, pear	hazelnut, walnut, almond, peanut, lentil, beans
**Cypress/Cedar**	peach, citrus fruit, apple, melon	tomato	
**Grass**	melon, watermelon, orange, tomato, kiwi	potato, swiss chard	peanuts
**Mugwort**	peach, lychee, mango, grape	celery, carrot, parsley, fennel, garlic, cabbage, broccoli, coriander, cumin	sunflower seeds, peanuts
**Pellitory**	peach, cherry, melon		pistachio
**Ragweed**	watermelon, banana	zucchini, cucumber, squash	
